# Mobile apps for treatment of speech disorders in children: An evidence-based analysis of quality and efficacy

**DOI:** 10.1371/journal.pone.0201513

**Published:** 2018-08-09

**Authors:** Lisa Furlong, Meg Morris, Tanya Serry, Shane Erickson

**Affiliations:** 1 Discipline of Speech Pathology, School of Allied Health, College of Science, Health and Engineering, La Trobe University, Melbourne, Australia; 2 Healthscope Northpark Private Hospital, Bundoora, Australia; 3 Centre for Sport and Exercise Medicine Research, School of Allied Health, La Trobe University, Melbourne, Australia; Kyoto University, JAPAN

## Abstract

**Background:**

Recently there has been exponential growth in mobile health (mHealth) applications (apps) for children with speech disorders. A challenge for health professionals and families is knowing how to find high quality apps that are therapeutically beneficial. We systematically search and critique the quality of mobile apps for childhood speech disorders. An evidence-based method for identifying suitable apps in the Google Play and Apple iTunes stores is also proposed.

**Methods and findings:**

A systematic search of the Google Play and Apple iTunes app stores was conducted from November 2016 to May 2017. Twelve pre-defined search terms were applied, identifying 5076 apps. Systematic screening resulted in 132 unique apps for full appraisal. These were appraised by two raters using the Mobile Application Rating scale. None were of excellent quality. Twenty-five were of good quality, 105 average and 2 were poor or very poor.

**Discussion:**

It can be challenging for consumers to locate high quality speech therapy apps for children. Although we found more than 5000 apps, less than 3% met criteria for evaluation. Difficulties sourcing valid apps included: (i) Boolean operators were not available and therefore only one search term could be used each time (ii) the order of app listings in online stores continually changed (iii) apps were organised in online stores according to relevance and popularity (iv) there was no easy way to extract app titles and eliminate duplicates (v) app cost did not always correlate with therapeutic quality.

**Conclusions:**

The rapid growth of mHealth heightens the need to develop rigorous and efficient systems to search and retrieve apps and evaluate their therapeutic benefits. Given the difficulty accessing speech therapy services worldwide, mHealth promises therapy benefits when apps are reliable, valid and easily found.

## Introduction

Speech disorders are common in children. These children are at risk of social and academic difficulties with persisting consequences into adolescence and adulthood [1, 2]. Early, effective and efficient speech therapy is important. For families, accessing speech therapy can be difficult due to workforce shortages of speech language pathologists [3, 4]. Cost, socioeconomic status and geographical location [5] can also be barriers. Technology-aided speech therapy and, in particular, mobile health (mHealth) has the potential to improve children’s access to speech therapy.

Mobile applications (apps) are accessible, affordable and engaging forms of healthcare. For example, it has been shown that asthma, bipolar disorder, obesity and chronic pain can be assisted by mobile apps that promote self-management [6] and optimise therapy adherence [7]. Apps can augment lifestyle interventions such as logging food intake and exercise [8]. For children with speech disorders, apps provide an opportunity for increased practice outside of usual clinical settings. This was demonstrated in a study comparing computer-led and parent-led home therapy for children with speech disorders [9]. Home practice time was higher for children in the computer-led mode and children and parents favoured this method. Apps also enable children to continue practising their speech therapy tasks at home, regardless of geographical location, socio-economic status or family circumstances.

Health professionals such as medical practitioners and allied health professionals can benefit from the affordability and accessibility of apps. Apps are located on mobile phones, computers or tablets which are usually durable and easy to transport. Some therapy apps can reduce health professionals’ preparation time and offer features such as automatic scoring and tracking of patient’s progress [10].

A large number of mobile apps are now available for children needing speech therapy. For children, families and health professionals, it can be hard to know which apps have therapeutic benefit. For consumers, there is an urgent need for trustworthy recommendations of clinically beneficial therapy apps for children with speech disorders.

In this article we provide a new method for searching and appraising mobile speech therapy apps for children. There were two main foci: (i) systematically searching and selecting apps, based on the Preferred Reporting Items for Systematic Reviews and Meta-Analyses (PRISMA) guidelines [11] and (ii) determining optimal methods for quality appraisal of mobile apps for childhood speech disorders using a valid and reliable tool.

The PRISMA guidelines (S1 Appendix) were adapted for the current study as some systematic review methods cannot be directly applied to commercial app stores or app content [12]. For example, search strings containing Boolean operators cannot be entered into app stores and as such, key words were entered individually.

## Method

The methods for this evaluation are detailed in a recent protocol publication by Furlong et al. [13].

### Locating mobile apps for childhood speech disorders

Apps for this review were identified via searching of two app stores, Google Play and Apple iTunes. These were chosen as they are linked to the two most widely-used operating platforms in the mobile market, Android and iOS.

Between the period November 2016 and May 2017, twelve predefined search terms were entered individually into the search fields of the web interface of the Google Play and Apple iTunes App stores: *‘speech*, *speech therapy*, *speech pathology*, *artic*, *articulation*, *speak*, *chat*, *say*, *talk*, *pronunciation*, *phonological*, *phonology*.*”* These terms were selected following consultation with practising speech language pathologists, allied health academics with an interest in childhood speech disorders and App Specialists at Apple Support and Google Play. In Apple iTunes, the 12 search terms were first entered for the iPad and then for the iPhone. App Specialists at Apple Support recommended this in order to capture all relevant apps. For Google Play, the 12 search terms were entered once into the search fields as there was no option to search by device compatibility.

Search results were returned in rows on the web interface for each of the app stores. Each row of apps was copied and pasted into a Microsoft Word [14] document, so that both the title and icon were available for later screening.

**Fig 1:**
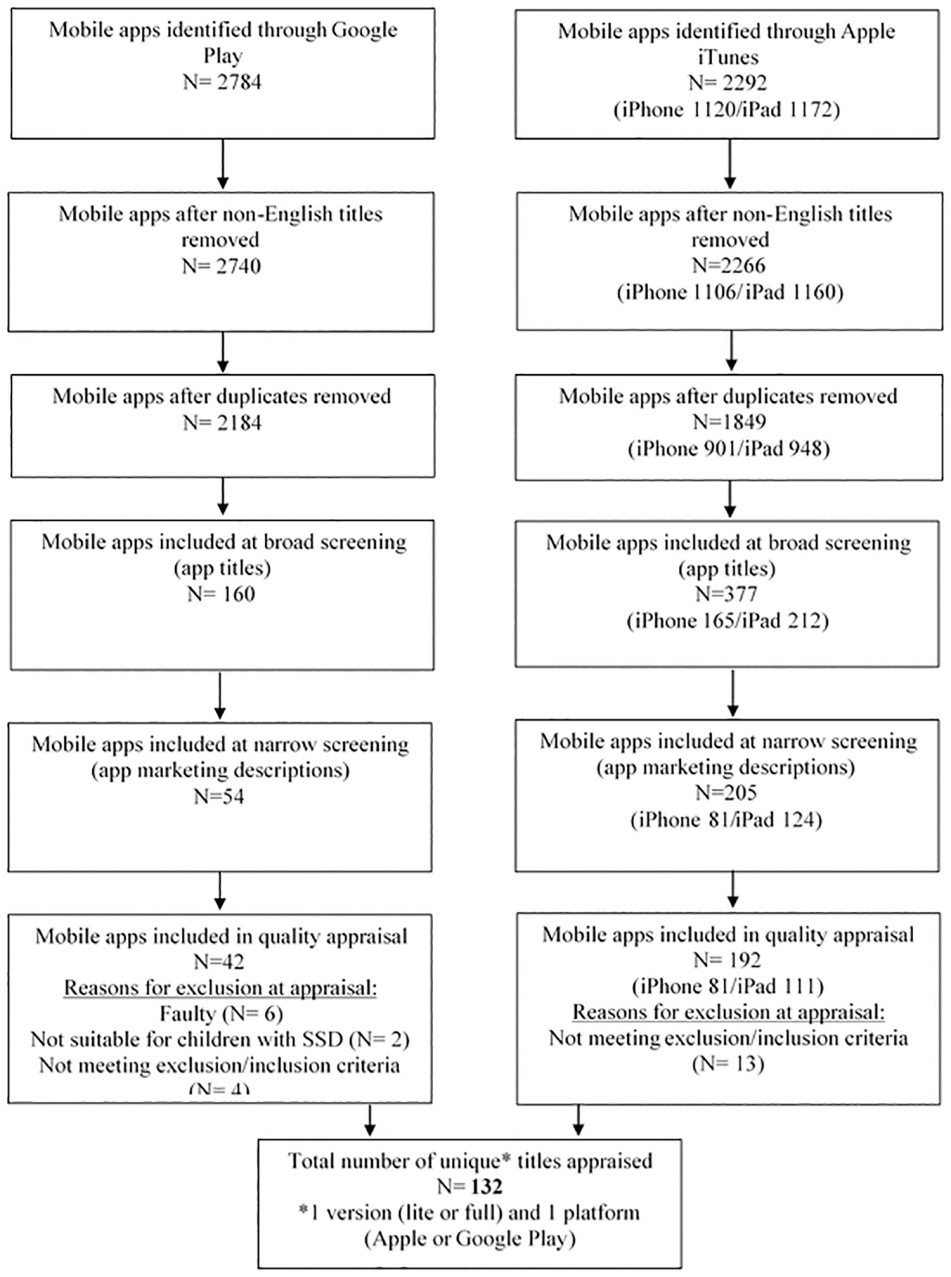
Systematic search and selection flow chart.

A total of 5076 titles were generated by the search. Google Play returned 2784 titles and Apple iTunes returned 2292 (1120 for iPhone and 1172 for iPad) (Fig 1). Titles were separated according to source: Google Play titles, Apple iTunes titles for iPhone and Apple iTunes titles for iPad. App icons could not be exported to Microsoft Excel [15], however were available for reviewers during the screening process when app titles were considered ambiguous for the purposes of determining eligibility for inclusion. Duplicate titles were manually removed by the first author. When the same title was found in both the iPhone and iPad lists, both were kept, to examine whether there were differences between the device versions. Non-English titles were also removed. Removal of duplicates and non-English titles left 4033 app titles for screening (refer to Fig 1).

### Selecting apps for quality appraisal

Two reviewers (the first author and a research assistant) were involved in the selection of apps for quality appraisal. Both were Certified Practising Speech Language Pathologists (of Speech Pathology Australia) with experience working with children with speech disorders as well as post-graduate research experience. Selection involved two phases- broad and focussed screening.

#### Phase one: Broad screening

Both reviewers independently screened all 4033 titles returned by the search. For inclusion, apps had to include activities or tasks that required production of speech by the user (i.e. not just listening or auditory discrimination tasks). They had to be running on Android or iOS and designed for speakers of English. Apps had to be suitable for children aged up to 12 years old with a speech disorder. Restricting the age to 12 was based on evidence from prevalence studies, suggesting that speech disorders are highly prevalent in preschool and school-aged children [16, 17]. Exclusion criteria were: apps providing speech production training for second language learners (including accent modification), apps for teaching foreign languages, speech to text/text to speech apps and alternative and augmentative communication apps. Apps designed for clients with voice disorders and developmental language disorders were also excluded as were apps providing assessment only.

Broad screening eliminated 3496 apps, resulting in a total of 537 apps for further screening (Fig 1).

#### Phase two: Focussed screening

Marketing descriptions were screened in phase two. The marketing descriptions of the 537 apps were extracted from the two stores. The same reviewers independently read the marketing descriptions for the 537 apps. The same inclusion and exclusion criteria were applied.

Inter-reviewer agreement based on Cohen’s Kappa statistic was 0.72. The strength of this agreement can be interpreted as “good” [18]. Disagreements were resolved by discussion until consensus was reached. Focussed screening resulted in a total of 259 apps for quality appraisal. A further 25 apps were excluded at quality appraisal, for reasons detailed in Fig 1.

### Comparing and appraising mobile apps for childhood speech disorders

#### Data extraction

Data extraction complied with the terms of service of Google Play and Apple iTunes. The following data were extracted from the Google Play and Apple iTunes stores for each of the 234 apps included for quality appraisal.

1. Version
2. Platform
3. Release date
4. Months since release
5. Number of updates
6. Average update frequency
7. Time of last update
8. Rating current version
9. Number of ratings for current version
10. Rating previous version
11. Number of ratings for previous version
12. Price for basic version
13. Price for upgrade or pro version
14. Device compatibility
15. Developer
16. Bundle option (yes/no)

To ensure accuracy in data extraction for these 16 items, the second reviewer involved in the screening process extracted data for 10% of the included apps (24 apps). Interrater reliability for data extraction was 92% for the 384 data points.

#### Quality appraisal

The 234 included apps were downloaded onto four devices for quality appraisal: a Samsung Galaxy Tab A 8.0 WiFi 16GB (Android Version 5.0 (Lollipop)), an iPad 3 (iOS version 10.2.1), a Samsung S3 (Android Version 4.3) and an iPhone 5S (iOS version 10.2.1). Using a tablet and a phone for each of the platforms allowed for comparison between tablet and phone versions of the included apps.

Quality was evaluated using the Mobile Application Rating Scale (MARS) [19]. This tool was designed by an expert multidisciplinary team at the Institute of Health and Biomedical Innovation and Queensland University of Technology. In the 2014 pilot of the MARS, high levels of interrater reliability were achieved. Moderate concurrent validity (with the app store star ratings) was also reported. The MARS evaluates apps using a 5-point scale (one = inadequate to five = excellent) across six indicators: 1) engagement (5 items), 2) functionality (4 items), 3) aesthetics (3 items), 4) information quality (7 items), 5) subjective quality (4 items) and 6) perceived impact (6 items). Response items for each indicator are listed in Table 1:

**Table 1:** MARS indicators and associated items.

Indicator	Response items
1. Engagement	Entertainment, interest, customisation, interactivity and target group.
2. Functionality	Performance, ease of use, navigation and gestural design.
3. Aesthetics	Layout, graphics and visual appeal.
4. Information quality	Accuracy of app description, goals, quality of information, quantity of information, visual information, credibility and evidence-base.
5. Subjective quality	Recommendation for app use, predicted usage over a 12-month period, willingness to pay for the app and overall star rating.
6. Perceived impact	Potential to improve the user’s awareness, knowledge, attitudes, help-seeking and motivation for change towards the health behaviour as well as the perceived behavioural change from use of the app.

Mean scores are calculated for each indicator to identify the strengths and weaknesses of the app. A total mean score is then calculated from the first four indicators. The highest possible total mean score is five. The developers of the MARS designed the tool in this way so that the total mean score can be directly converted to a star rating, for comparison with app store star ratings. For example, a total mean score of 4.5 according to the MARS is equivalent to an app store star rating of 4.5. The recommendations provided by the MARS developers for use of the scale were followed throughout the appraisal process, including viewing of an online video tutorial.

The 234 mobile apps included for quality appraisal were trialled and rated independently by the same two reviewers involved in selection and screening. Each reviewer trialled each app for a total of 10 minutes. An additional five minutes was allocated to allow for comparison between devices and versions (e.g. lite/full) when applicable. After comparison between lite/full versions and phone/tablet versions, 36 unique app titles were appraised in Google Play and 103 in Apple iTunes. With the two app stores combined, 132 unique titles were identified and appraised. The results of the MARS for the 132 unique titles are provided in S2 Appendix.

In accordance with the MARS guidelines, the total mean score was calculated for each app based on the first four MARS indicators (engagement, functionality, aesthetics and information quality). The scores for each app were then averaged for the two reviewers (see ranking S3 Appendix). Item four of the Subjective Quality indicator of the MARS asks: “what is your overall star rating for this app?” Each app was therefore allocated a subjective star rating by each reviewer. An average star rating was then calculated for each of the 132 apps, based on the reviewers’ star ratings (S3 Appendix).

A two-way mixed ICC based on the total MARS score, was used to determine how consistent the two raters were. An ICC of .72 (95% CI 0.60–0.80) was achieved indicating satisfactory interrater reliability [20]. Interrater reliability for each of the four indicators comprising the total MARS score are as follows: engagement ICC .77 (95% CI 0.67–0.84); functionality ICC 0.49 (95% CI 0.29–0.64); aesthetics ICC 0.72 (95% CI 0.61–0.80) and; information quality ICC 0.55 (95% CI 0.36–0.68).

## Results

### General characteristics of reviewed apps

Descriptive data are summarised in Table 2. Individual characteristics are presented in S3 Appendix. Apple iTunes returned a higher number of relevant titles than Google Play. The majority of apps across both app stores were paid apps. More apps in Google Play had star ratings than Apple iTunes. Of the 19 with star ratings in Google Play, 12 (63.2%) were reviewed by five consumers or less and seven (37%) were reviewed by one consumer. Reviewers are not identified and therefore it is difficult to determine the credibility of the average star rating, particularly for apps with less than five reviews. In Apple iTunes, it is a requirement that an app receive at least five reviews before an average star rating is calculated. This is likely to contribute to fewer apps in Apple iTunes having a star rating.

**Table 2:** Key descriptive data for the reviewed apps.

	**Platform/total number of app titles**	**Number of apps with an update history**	**Median update frequency** **(in months)**	**Range for update frequency** **(in months)**
Update frequency in months	Apple iTunes/103	84 (81.6%)	9.7	1–77
	Google Play/36	Not provided		
	**Platform/ total number of app titles**	**Number of apps with an app store star rating**	**Median star rating (out of 5) for rated apps**	**Star rating range for rated apps (star rating out of 5)**
App store star rating	Apple iTunes/103	2 (1.9%)	3.5 (based on an average of 10 (SD 7.1) consumer ratings per rated app)	3–4
	Google Play/36	19 (52.8%)	3.1 (based on an average of 31.1 (SD 79) consumer ratings per rated app)	1–5
	**Platform/ total number of app titles**	**Number of full-version apps**	**Median price of full-version apps**	**Price range for full-version apps**
Full-version apps: price (AUS$)	Apple iTunes/103	103 (100%)	$14.99	$0.00-$279.99
	Google Play/36	36 (100%)	$3.89	$0.00- $86.13
Developed by SLP	Apple iTunes/103	81 (78.6%)	-	-
	Google Play/36	29 (81%)	-	-

Version history, including number and time of updates, was provided only by Apple iTunes. Without a version history, it is difficult to know how an app will function on newer versions of software or whether identified bugs have been fixed prior to purchase. In both stores, approximately 80% of the reviewed apps had been developed by a speech language pathologist or by a team inclusive of a speech language pathologist.

### Descriptive data for the reviewed apps

#### App quality

A large proportion of the reviewed apps were of average to good quality as measured by the MARS. None achieved a total MARS score of five (excellent). Detailed results of the MARS appraisal are provided in S2 Appendix.

Functionality was the highest-scoring MARS indicator. There were six faulty Google Play apps removed at quality appraisal. These apps did not open or crashed within the first minute of use. No faulty Apple iTunes apps were identified. Aesthetics and information were the next two highest-scoring indicators, followed by engagement. Apple iTunes achieved higher overall mean scores than Google Play across three indicators. Apple iTunes and Google Play apps scored equally for information quality.

The average MARS score for the reviewed apps was 3.7 (*SD = 0*.*3*) and ranged from 2.5 to 4.6 out of 5. The number of apps achieving a total MARS score of 4–4.9 was 25. Therefore of the 132 mobile apps, 25 (19%) were considered to be of good quality. The majority (N = 105 or 80%) achieved a total MARS score of 3–3.9, indicating that most apps for children with speech disorders were considered of average quality. Two apps (1.5%) achieved a total MARS score of less than three.

The top five apps based on the average total MARS score were: Articulation Station Pro (4.6), Apraxia RainbowBee (4.5), Articulation Scenes (4.5), Speech with Milo Articulation Board Game Pro (4.4) and Articulate it! Pro (4.4). These top five apps ranged in price from $29.99 to $89.99 (AUD). Four apps received a star rating of five: Articulation Station Pro, Articulation Scenes, Speech with Milo Articulation Board Game and, Articulate it Pro. These apps provided numerous options for customisation including the ability to choose the position of the phoneme in the target words, the syllable structure of the target words and the level of linguistic complexity. Based on the reviewers’ ratings, these apps were perceived to be aesthetically appealing judged by the use of high resolution images; professional, clear and stylistically consistent layouts; use of colour to enhance menus and app features and; adequate sizing and arrangement of app buttons. Content was thought by the reviewers to be displayed in engaging and novel ways. There were engaging design features such as board games, memory, finding hidden items in scenes, guessing games and stories. The authors do acknowledge that this judgement may vary across users.

The behaviour change scale of the Perceived Impact indicator of the MARS required reviewers to rate the following statement on a 5-point Likert scale (1 = strongly disagree→5 = strongly agree): “the use of this app is likely to increase/decrease (insert target health behaviour):” Therefore for this study, the behaviour change scale of the Perceived Impact indicator of the MARS was used to determine the potential of the reviewed apps to increase speech production accuracy or to reduce speech sound production errors. To score this scale, reviewers identified the underlying principles of intervention of the app (e.g. phonology, speech perception, motor learning) and the app’s intervention procedures (e.g. antecedents, stimulus, user response, consequent events/feedback and activities). Overall, the average behaviour change score was 3.2 (*SD = 0*.*7*). There were 19 apps rated between four and five indicating that only 14% were considered to have potential therapeutic benefit for children with speech disorders. A description of the therapeutic features of these 19 apps in relation to intervention principles and procedures is provided in S4 Appendix.

Therapeutic benefit was often compromised by therapy exemplars (i.e. the target words for therapy). Vocabulary was sometimes inappropriate for children, for example, the word “mi**sn**omer” to target medial /sn/. Therapy exemplars could not always be filtered by syllable structure and as such, multisyllabic words were often included. When exemplars could be filtered by the position of the phoneme in the words, the phoneme would often appear more than once in any given word. For example, “**c**a**k**e” for initial /k/, where /k/ is in both word initial and final position. In one app, phonemes were offered in positions which are not permissible in English, e.g. initial /ŋ/.

Modelling of the therapy exemplars was sometimes incorrect or difficult to understand. For practice with isolated phonemes, the schwa (the neutral vowel in unstressed syllables) was sometimes inserted after the target phoneme. Short vowels were sometimes unnaturally prolonged. Many apps used natural speech to model the therapy exemplars, however one used synthetic speech which was difficult to understand. In one, words within the minimal pairs were not always true minimal pairs (depending on English dialect). For example, for /r/ and /w/, the words were “roof” and “woof” (/r**ʊ**f/w**ʊ**f/) and “rock” and “walk” (/r**ɒ**k/w**ɒ**k/). Some apps offered the option of breaking the word into its phonemes for practice. For one, this was modelled incorrectly, e.g. /k**ɒr**n/ rather than /k**ɔ**n/. Many of the apps offered practise with the target phoneme or phonological process at carrier phrase, sentence or story level. In one, the carrier phrase “I keep” was used, however this did not make syntactic or semantic sense for many of the target words, e.g. “I keep bake, I keep raccoon” (for the /k/ sound).

## Discussion

This analysis identified 132 unique mobile app titles for use with children with speech disorders. These apps appear best suited to children with speech disorders characterised by difficulties with speech perception, phonological representations, articulation/motor production and phonotactics. The reviewed apps scored highly for functionality and were overall rated as average across the remaining three indicators of the MARS. Only a small proportion were considered to be of very high quality or therapeutically beneficial.

The process of searching the app stores was challenging. Boolean operators could not be used, therefore only one search term could be entered each time. The resulting titles had to be extracted immediately following the search and in their entirety. This was due to the order of app listings in the stores. This changes continually, based on relevance and popularity. For this review, this system of listing also meant that a relevant title could be returned in one store but not another. Similarly, a relevant title could be returned on one device (e.g. iPad) but not another (e.g. iPhone). As a result, a process of checking platform and device compatibility for the included apps had to be created. Additional relevant apps were identified during the data extraction phase. The way apps are titled, described and assigned key words by developers means that relevant apps can sometimes be missed.

Extracting titles from the apps stores was challenging for a number of reasons. App titles and icons had to be extracted using screenshots. Apple iTunes prevents copying of marketing descriptions from the store. For this reason, each title had to be individually entered into a web browser followed by ‘itunes.com.’ This method was used to find, extract and collate marketing descriptions in a central location for screening. There is no easy way to remove duplicate titles within the app stores. Removal of duplicate titles was conducted manually in Microsoft Excel [15]. App bundles in Apple iTunes made duplicate identification difficult. Across both platforms, apps were sometimes labelled inconsistently. The app name in the app store did not always match the name of the app once it was downloaded onto a device. App titles for a single app were sometimes different between the two app stores. For example, Articulation R & R Blends on Apple iTunes was named Articulation R Flashcards on Google Play.

The total MARS score describes the overall quality of an app which does not necessarily correlate with therapeutic benefit. An app can achieve a high total MARS score assuming it functions adequately, is engaging and aesthetically pleasing; however, it may not always be clinically useful. For this reason, analysis of the therapeutic features of the included apps was conducted using the behaviour change scale of the Perceived Impact indicator of the MARS, to consider the potential of the included apps to increase speech sound production accuracy or to reduce speech sound production errors. An appraisal tool with a greater focus on the therapeutic benefits of mobile apps is needed. There is a need to comprehensively and objectively evaluate a variety of constructs relating to evidence-based treatment strategies and principles, as well as an app’s potential for behaviour change. For children with speech disorders, it would be helpful to evaluate the underlying principles of intervention and the intervention procedures. Information generated by such a tool would support consumers in making informed decisions when engaging in mobile health.

Mobile health is becoming increasingly popular. Consumers are looking to mobile apps as an accessible, affordable and engaging way to manage and promote their health. It is currently difficult for consumers to identify mHealth apps which are of high quality and of therapeutic benefit. Key word searching using the store search engines may not always identify the most relevant products. High quality apps are sometimes obscured by a large number of irrelevant or low quality ones. Ambiguous titles, broad marketing descriptions and reviews from potentially questionable sources mean that consumers are sometimes at risk of purchasing apps which might be of little therapeutic value. While this review is specific to mobile apps for speech therapy in children, other studies have also identified a mismatch between the commercial and scientific domains of mHealth [21]. This raises an ethical issue around the quality of treatment and care that consumers of mHealth are receiving. The use of mobile health apps also raises privacy issues for consumers given that some collect, store and use personal information.

Mobile health has the potential to improve access to high quality therapeutic tools that support and promote health. For children with speech disorders, it has the potential to improve communication. Future research should consider ways to integrate mHealth and evidence-based practice to facilitate the development of high quality, clinically beneficial tools. A process of screening and regulating mobile health apps within the online stores is needed. Improved search engines are also necessary so that consumers can easily access relevant and beneficial apps.

## Limitations

The review only reports on apps available at the time of the search. The authors acknowledge that new ones may have been released since this time of the search. The review reports only on English language apps. The feasibility of using the reviewed apps with children with physical and intellectual disabilities was beyond the scope of the review. Consumers may consider the information presented in this review alongside the individual needs of clients and/or children when making decisions regarding app suitability. Additionally, the authors acknowledge that appropriate selection and use of an app will depend on the prior knowledge and skills of consumers.

The authors were unable to provide alternatives for automatic data extraction or analysis in relation to mobile app data. No automated processes appear to exist. This could be an area for future development. The therapeutic benefit of the included apps was determined using the behaviour change scale of the Perceived Impact indicator of the MARS. It is not known whether this score correlates with the actual change observed in children’s speech production, as a result of using these apps.

## Supporting information

S1 Appendix PRISMA 2009 checklist. (DOC)

S2 Appendix Complete MARS appraisal. (DOCX)

S3 Appendix Individual app characteristics by rank order. (DOCX)

S4 Appendix Therapeutic features of top 19 apps for behaviour change. (DOCX)
