# Supplementary material for: Mobile apps for treatment of speech disorders in children: An evidence-based analysis of quality and efficacy
Source: PLoS One. 2018 Aug 9;13(8):e0201513. doi: 10.1371/journal.pone.0201513 (PMC6084897; doi:10.1371/journal.pone.0201513)
Supplement: S2 Appendix — (DOCX) [file pone.0201513.s002.docx]

**S2 Appendix: Complete MARS appraisal**

|  | **SECTION A:**  **ENGAGEMENT**  **MEAN SCORE** | | **SECTION B:**  **FUNCTIONALITY**  **MEAN SCORE** | | **SECTION C:**  **AESTHETICS**  **MEAN SCORE** | | **SECTION D:**  **INFORMATION QUALITY**  **MEAN SCORE** | | **TOTAL MARS SCORE** | | |
| --- | --- | --- | --- | --- | --- | --- | --- | --- | --- | --- | --- |
| **APP** | **RATER 1** | **RATER 2** | **RATER 1** | **RATER 2** | **RATER 1** | **RATTER 2** | **RATER 1** | **RATER 2** | **RATER 1** | **RATER 2** | **DIFF** |
| APRAXIA-EARLY INTERVENTION 1 | 1.6 | 2.6 | 1.5 | 3 | 2 | 3.3 | 2.3 | 3 | **1.9** | **3** | 1.1 |
| ALL ABOUT SOUNDS HD-INITIAL POSITION WORDS LITE | 3 | 2.8 | 4.3 | 4.3 | 3.3 | 4 | 3.3 | 3.7 | **3.5** | **3.7** | 0.2 |
| APRAXIA PICTURE SOUND CARDS APSC | 3.8 | 3.8 | 4 | 4 | 4.3 | 4.3 | 4.2 | 4.2 | **4.1** | **4.1** | 0 |
| APRAXIA RAINBOWBEE | 4.4 | 4.4 | 4.5 | 4.5 | 5 | 4.7 | 4.2 | 4 | **4.5** | **4.4** | 0.1 |
| APRAXIMATIONS | 2.6 | 2.8 | 4.3 | 4.5 | 4 | 4 | 2.3 | 3.7 | **3.3** | **3.8** | 0.5 |
| ARTIC PHOTOS ‘L’ FUN DECK PLUS | 3 | 2.6 | 4.3 | 4.5 | 4 | 4 | 4 | 3.7 | **3.8** | **3.7** | 0.1 |
| ARTIC PHOTOS ‘R’ FUN DECK PLUS | 3 | 2.6 | 4.3 | 4.5 | 4 | 4 | 4 | 3.7 | **3.8** | **3.7** | 0.1 |
| ARTIC PHOTOS ‘S’ FUN DECK PLUS | 3 | 2.6 | 4.3 | 4.5 | 4 | 4 | 4 | 3.7 | **3.8** | **3.7** | 0.1 |
| ARTIC PRACTICE | 3.8 | 2.8 | 3.8 | 4.5 | 2.7 | 3.7 | 3.4 | 3.3 | **3.4** | **3.6** | 0.2 |
| ARTICULATE IT! PRO | 4 | 4.8 | 4.3 | 4.5 | 4.7 | 4.7 | 4 | 4 | **4.3** | **4.5** | 0.2 |
| ARTICULATION CARNIVAL PRO | 4 | 4 | 4.8 | 4.8 | 4.3 | 4.3 | 4 | 3.7 | **4.3** | **4.2** | 0.1 |
| ARTICULATION COACH-L | 2.4 | 2.2 | 4.5 | 4.5 | 4 | 4.3 | 3 | 3.3 | **3.5** | **3.6** | 0.1 |
| ARTICULATION COACH-P | 2.4 | 2.2 | 4.5 | 4.5 | 4 | 4.3 | 3 | 3.3 | **3.5** | **3.6** | 0.1 |
| ARTICULATION COACH-R | 2.4 | 2.2 | 4.5 | 4.5 | 4 | 4.3 | 3 | 3.3 | **3.5** | **3.6** | 0.1 |
| ARTICULATION FLASH CARDS /R/ | 2.6 | 2.8 | 4 | 4.5 | 3.7 | 3.7 | 3.7 | 3.7 | **3.5** | **3.7** | 0.2 |
| ARTICULATION FLIP BOOKS FREE | 3.4 | 3.2 | 3.8 | 5 | 4.3 | 4 | 3.7 | 3.7 | **3.8** | **4** | 0.2 |
| ARTICULATION GAME MDB | 2.8 | 3 | 5 | 4.8 | 4 | 4.7 | 2.7 | 3 | **3.6** | **3.9** | 0.3 |
| ARTICULATION GAMES | 4.4 | 4 | 3.8 | 4.8 | 4.3 | 4.3 | 4 | 3.7 | **4.1** | **4.2** | 0.1 |
| ARTICULATION ISLAND | 3 | 3.2 | 2.8 | 3 | 1.3 | 2.7 | 3 | 3.3 | **2.5** | **3.1** | 0.6 |
| ARTICULATION IV | 3.8 | 2.8 | 3.8 | 4.5 | 2.7 | 3.7 | 3.4 | 3.3 | **3.4** | **3.6** | 0.2 |
| ARTICULATION LITE WITH THE SPEECH WIZARD | 3.6 | 3.8 | 3.8 | 4.3 | 3.7 | 4.7 | 3.4 | 4 | **3.6** | **4.2** | 0.6 |
| ARTICULATION R & R BLENDS | 2.8 | 3 | 5 | 4.8 | 4 | 4.7 | 2.7 | 3 | **3.6** | **3.9** | 0.3 |
| ARTICULATION SCENES | 3.8 | 4.6 | 4.5 | 4.8 | 5 | 5 | 4 | 4 | **4.3** | **4.6** | 0.3 |
| ARTICULATION STATION PRO | 4.4 | 4.6 | 5 | 4.8 | 4.7 | 4.7 | 4.4 | 4.4 | **4.6** | **4.6** | 0 |
| ARTICULATION VACATION | 4.2 | 4.4 | 4 | 3.8 | 5 | 5 | 4 | 4 | **4.3** | **4.3** | 0 |
| ARTIKPIX | 3.4 | 3.2 | 4.8 | 4.5 | 3.3 | 4 | 4 | 3.7 | **3.9** | **3.9** | 0 |
| ARTIKPIX LEVELS | 4 | 2.8 | 4.3 | 4.3 | 3.7 | 4.3 | 4 | 3.3 | **4** | **3.7** | 0.3 |
| BIGMOUTH SOUNDS | 1.8 | 1.8 | 4 | 4 | 3.7 | 3.7 | 3.3 | 3.3 | **3.2** | **3.2** | 0 |
| CHARADES ARTICULATION FOR SPEECH THERAPY | 3.4 | 3 | 4.8 | 4.8 | 3.7 | 4.3 | 3.7 | 3.3 | **3.9** | **3.9** | 0 |
| CLUSTERS COMPLEX LITE | 3.8 | 3.2 | 4.5 | 3.8 | 4 | 4 | 3.8 | 3.7 | **4** | **3.7** | 0.3 |
| DANCE PARTY ARTICULATION FOR SPEECH THERAPY | 3.4 | 3.2 | 4.5 | 4.5 | 4 | 4 | 3.3 | 3.3 | **3.8** | **3.8** | 0 |
| DIADO SYLLABLES | 2.6 | 2.6 | 4.5 | 4.5 | 3 | 4 | 3 | 3.3 | **3.3** | **3.6** | 0.3 |
| DRILLABY PRO SPEECH THERAPY GAME-SLP EDITION | 3.6 | 3.2 | 4.5 | 3 | 3.3 | 4.3 | 4 | 3.7 | **3.9** | **3.6** | 0.3 |
| DRILLABY -FAMILY EDITION | 3.6 | 3.2 | 4.5 | 3 | 3.3 | 4.3 | 4 | 3.7 | **3.9** | **3.6** | 0.3 |
| ELR-OFFLINE FOR IPAD | 3.2 | 3.2 | 4.5 | 4.5 | 3.7 | 3.7 | 3.7 | 3.7 | **3.8** | **3.8** | 0 |
| FLEXIBLE SPEECH | 2.4 | 3 | 4.5 | 4.8 | 4.3 | 4 | 3.3 | 3.3 | **3.6** | **3.8** | 0.2 |
| FLEXIBLE SPEECH USA | 2.4 | 3 | 4.5 | 4.8 | 4.3 | 4 | 3.3 | 3.3 | **3.6** | **3.8** | 0.2 |
| FRICATIVES ARTICULATION | 2.8 | 2.8 | 4.5 | 4.8 | 4 | 4.7 | 3.3 | 3 | **3.7** | **3.8** | 0.1 |
| FUN WITH R | 3.8 | 3.8 | 4.3 | 4.5 | 4.3 | 4.3 | 4 | 4 | **4.1** | **4.2** | 0.1 |
| GAME SHOW ARTICULATION FOR SPEECH THERAPY | 3 | 3.2 | 4.8 | 4.8 | 4.3 | 4 | 3 | 3.7 | **3.8** | **3.9** | 0.1 |
| GHK ARTICULATION | 2.8 | 2.8 | 5 | 4.8 | 3.7 | 4.7 | 3 | 3 | **3.6** | **3.8** | 0.2 |
| GUESS THE PICTURE FOR ARTICULATION PRACTICE (CH, SH) | 3.4 | 2.6 | 3.8 | 4 | 3.3 | 3.7 | 3.7 | 3.3 | **3.6** | **3.4** | 0.2 |
| GUESS THE PICTURE FOR ARTICULATION PRACTICE (K, G) | 3.4 | 2.6 | 4 | 4 | 3.3 | 3.7 | 3.7 | 3.3 | **3.6** | **3.4** | 0.2 |
| HIGH FREQUENCY WORDS FOR SPEECH THERAPY- FOR SPEECH THERAPY | 2.4 | 2.4 | 4.3 | 4.8 | 4 | 3.3 | 3.7 | 3.7 | **3.6** | **3.6** | 0 |
| I CAN ARTICULATE | 2.4 | 2.4 | 4.8 | 4.8 | 3.7 | 3.7 | 3.3 | 3.3 | **3.6** | **3.6** | 0 |
| I DARE YOU ARTICULATION FOR SPEECH THERAPY | 3.2 | 2.8 | 4.8 | 4.8 | 3.7 | 4.3 | 3.3 | 3.3 | **3.8** | **3.8** | 0 |
| JUNGANEW: A HERD OF SOUND S ‘S’ FREE | 3.4 | 3.2 | 3 | 4 | 4.7 | 4 | 3 | 3.3 | **3.5** | **3.6** | 0.1 |
| KIDS SOUND LAB- D SOUND OF THE WOODPECKER | 4 | 3.4 | 4.3 | 4.5 | 3.7 | 4.3 | 4.3 | 3.8 | **4.1** | **4** | 0.1 |
| L & L BLENDS | 2.8 | 2.8 | 4.5 | 4.8 | 4 | 4.7 | 3.3 | 3 | **3.7** | **3.8** | 0.1 |
| LEXICO ARTICULATION | 4 | 3 | 4.3 | 4.3 | 4.3 | 4 | 4 | 3.3 | **4.2** | **3.7** | 0.5 |
| LINGUISYSTEMS PHONOLOGY CARDS | 3.6 | 3 | 3.8 | 4.5 | 3.3 | 4 | 4 | 3.3 | **3.7** | **3.7** | 0 |
| LISP THERAPY FREE | 3.2 | 3 | 3 | 4.5 | 2.7 | 4 | 2.5 | 3.7 | **2.9** | **3.8** | 0.9 |
| LISTEN CLOSE ARTICULATION FOR SPEECH THERAPY | 3.4 | 2.8 | 4.3 | 4.8 | 3.7 | 4.3 | 3 | 3.3 | **3.6** | **3.8** | 0.2 |
| MINIMAL PAIRS ACADEMY | 3.4 | 4 | 4 | 4.5 | 3.3 | 4.7 | 4 | 4 | **3.7** | **4.3** | 0.6 |
| MINIMAL PAIRS FOR SPEECH THERAPY | 3.2 | 3 | 5 | 4.3 | 3.7 | 3.7 | 3 | 3.7 | **3.7** | **3.7** | 0 |
| MINIMAL PAIRS (LEARNING FUNDAMENTALS) | 2.6 | 2.8 | 3.5 | 4.5 | 2.3 | 3.7 | 3.3 | 3.7 | **2.9** | **3.7** | 0.8 |
| MINIMAL PAIRS (THERAPY BOX LIMITED) | 3.6 | 3.6 | 4.8 | 4.5 | 3.7 | 4.3 | 3.3 | 3.7 | **3.9** | **4** | 0.1 |
| MISSING LETTER ARTICULATION FOR SPEECH THERAPY | 3.2 | 2.8 | 4.8 | 4.8 | 3.7 | 4.3 | 3.7 | 3.3 | **3.9** | **3.8** | 0.1 |
| MULTIPLE CHOICE ARTICULATION FOR SPEECH THERAPY | 3.2 | 2.8 | 4.8 | 4.8 | 3.7 | 4.3 | 3.3 | 3.3 | **3.8** | **3.8** | 0 |
| MY ARTICULATION: INITIAL K | 3.6 | 3.4 | 4.3 | 4.8 | 4.3 | 4.3 | 3 | 3.3 | **3.8** | **4** | 0.2 |
| MYARTIC | 2.6 | 3.4 | 2.5 | 4.5 | 2.3 | 3.7 | 3 | 3.7 | **2.6** | **3.8** | 1.2 |
| OPEN-ENDED ARTICULATION FOR SPEECH THERAPY | 3.2 | 2.8 | 4.8 | 4.8 | 3.7 | 4.3 | 3.3 | 3.3 | **3.8** | **3.8** | 0 |
| PHONICS STUDIO | 3 | 3.2 | 4.8 | 4.5 | 3.3 | 4.3 | 3.3 | 3.7 | **3.6** | **3.9** | 0.3 |
| PHONOLOGICAL PROCESSES | 4 | 3.8 | 3.8 | 4.8 | 5 | 4.3 | 4.2 | 4 | **4.3** | **4.2** | 0.1 |
| PHONOLOGY | 2.6 | 2.8 | 3.5 | 4.5 | 2.3 | 3.7 | 3.3 | 3.7 | **2.9** | **3.7** | 0.8 |
| PHONOLOGY MATCHUPS! | 3 | 2.8 | 3 | 4.5 | 3 | 3.7 | 3.2 | 3.7 | **3.1** | **3.7** | 0.6 |
| PHONOPIX-FULL | 2.8 | 3 | 3.8 | 4.5 | 2.7 | 4 | 3.7 | 3.7 | **3.3** | **3.8** | 0.5 |
| POCKET ARTIC | 3.4 | 3.2 | 3.5 | 4.5 | 3 | 3.7 | 3.7 | 3.7 | **3.4** | **3.8** | 0.4 |
| POCKET PAIRS | 3.2 | 3.2 | 4.3 | 4.5 | 2.7 | 3.7 | 3.7 | 3.7 | **3.5** | **3.8** | 0.3 |
| PUPPY MINIMAL PAIRS | 2.8 | 3 | 5 | 5 | 4.3 | 4 | 3.3 | 3.7 | **3.9** | **3.9** | 0 |
| QUICK ARTIC | 2.4 | 3 | 3.5 | 5 | 3.7 | 4 | 3 | 3.7 | **3.2** | **3.9** | 0.7 |
| R ARTICULATION | 2.8 | 2.8 | 5 | 4.8 | 3.7 | 4.7 | 3 | 3 | **3.6** | **3.8** | 0.2 |
| R INTENSIVE PRO | 4 | 3 | 4.8 | 4.8 | 4 | 4.3 | 4 | 3.7 | **4.2** | **4** | 0.2 |
| S&S BLENDS ARTICULATION | 2.8 | 2.8 | 5 | 4.8 | 3.7 | 3.7 | 3 | 3 | **3.6** | **3.6** | 0 |
| SCIP (SOUND CONTRASTS IN PHONOLOGY) | 3.6 | 3.2 | 4.3 | 4.5 | 4 | 3.7 | 4.3 | 4 | **4.1** | **3.9** | 0.2 |
| S, Z, & S BLENDS | 2.8 | 2.8 | 4.5 | 4.8 | 4 | 4.7 | 3.3 | 3 | **3.7** | **3.8** | 0.1 |
| SECRET MISSION ARTICULATION FOR SPEECH THERAPY | 3.2 | 3 | 4.8 | 4.8 | 4 | 4.3 | 3.3 | 3.3 | **3.8** | **3.9** | 0.1 |
| SH CH ARTICULATION | 2.8 | 2.8 | 5 | 4.8 | 3.7 | 4.7 | 3 | 3 | **3.6** | **3.8** | 0.2 |
| SILLY SENTENCE ARTICULATION FOR SPEECH THERAPY | 3.2 | 3 | 4.8 | 4.8 | 3.7 | 4.3 | 3.3 | 3.3 | **3.8** | **3.9** | 0.1 |
| SLP MINIMAL PAIRS LITE-TOOL FOR SPEECH THERAPY | 2.8 | 3.2 | 2.5 | 4.5 | 2.3 | 4 | 3.7 | 4 | **2.8** | **3.9** | 1.1 |
| SPEECH CARDS BY TEACH APPS- FOR SPEECH THERAPY | 2.6 | 3 | 4.8 | 4.8 | 3 | 4 | 3 | 3.7 | **3.4** | **3.9** | 0.5 |
| SPEECH CARDS LITE | 2.6 | 2.6 | 3.3 | 4.5 | 3 | 4 | 2.3 | 3.3 | **2.8** | **3.6** | 0.8 |
| SPEECH CORNERS | 2.8 | 3 | 4.5 | 4.5 | 2.7 | 3.7 | 3.3 | 3.7 | **3.3** | **3.7** | 0.4 |
| SPEECH ESSENTIALS THERAPY APP | 3.6 | 4 | 4.3 | 4.8 | 4 | 4.7 | 4 | 4 | **4** | **4.4** | 0.4 |
| SPEECH FLIPBOOK- ARTICULATION & APRAXIA | 3.4 | 2.8 | 4.3 | 4.5 | 4 | 4 | 3.3 | 3.3 | **3.8** | **3.7** | 0.1 |
| SPEECH HANGMAN | 2.8 | 3 | 4.5 | 4.5 | 2.7 | 3.7 | 3.3 | 3.7 | **3.3** | **3.7** | 0.4 |
| SPEECH SOUNDS FOR KIDS- AUSTRALIAN EDITION | 4.4 | 2.8 | 3.8 | 4 | 3.3 | 3.7 | 4.2 | 3.3 | **3.9** | **3.5** | 0.4 |
| SPEECH SOUNDS FOR KIDS LITE-US EDITION | 4.4 | 2.8 | 3.8 | 4 | 3.3 | 3.7 | 4.2 | 3.3 | **3.9** | **3.5** | 0.4 |
| SPEECH SOUNDS ON CUE FOR IPAD LITE (AUS ENGLISH) | 3.2 | 2.2 | 3.5 | 4.8 | 3.7 | 3.7 | 3.3 | 3.3 | **3.4** | **3.5** | 0.1 |
| SPEECH SQUARES | 3.2 | 3 | 3.8 | 4.5 | 2.7 | 3.7 | 3.3 | 3.7 | **3.3** | **3.7** | 0.4 |
| SPEECH THAT WORKS | 3.4 | 4.4 | 3.5 | 4.8 | 3 | 4.7 | 3.6 | 4 | **3.4** | **4.5** | 1.1 |
| SPEECH THERAPY, APRAXIA ARTICULATION WORD FLASHCARDS | 2.4 | 3 | 3.8 | 4.2 | 2.7 | 3.7 | 2 | 3.3 | **2.7** | **3.6** | 0.9 |
| SPEECH THERAPY: B | 3 | 2.2 | 4 | 4.5 | 2.7 | 3.7 | 3.4 | 4.2 | **3.3** | **3.7** | 0.4 |
| SPEECH THERAPY CENTER | 3.6 | 2.8 | 4 | 3.8 | 4.3 | 3.7 | 3.7 | 3.3 | **3.9** | **3.4** | 0.5 |
| SPEECH THERAPY: CH | 3 | 2.2 | 4 | 4.5 | 2.7 | 3.7 | 3.4 | 4.2 | **3.3** | **3.7** | 0.4 |
| SPEECH THERAPY: D | 3 | 2.2 | 4 | 4.5 | 2.7 | 3.7 | 3.4 | 4.2 | **3.3** | **3.7** | 0.4 |
| SPEECH THERAPY: F | 3 | 2.2 | 4 | 4.5 | 2.7 | 3.7 | 3.4 | 4.2 | **3.3** | **3.7** | 0.4 |
| SPEECH THERAPY FOR APRAXIA-2 SYLLABLE WORDS | 3 | 3 | 4.5 | 3.5 | 3.7 | 4 | 3.2 | 3.3 | **3.6** | **3.5** | 0.1 |
| SPEECH THERAPY FOR APRAXIA –NACD SPEECH THERAPIST | 3 | 3 | 4.5 | 3.5 | 3.7 | 4 | 3.2 | 3.3 | **3.6** | **3.5** | 0.1 |
| SPEECH THERAPY FOR APRAXIA- WORDS | 3 | 3 | 4.5 | 3.5 | 3.7 | 4 | 3.2 | 3.3 | **3.6** | **3.5** | 0.1 |
| SPEECH THERAPY FOR APRAXIA-ENDINGS | 3 | 3 | 4.5 | 3.5 | 3.7 | 4 | 3.2 | 3.3 | **3.6** | **3.5** | 0.1 |
| SPEECH THERAPY: G | 3 | 2.2 | 4 | 4.5 | 2.7 | 3.7 | 3.4 | 4.2 | **3.3** | **3.7** | 0.4 |
| SPEECH THERAPY: J | 3 | 2.2 | 4 | 4.5 | 2.7 | 3.7 | 3.4 | 4.2 | **3.3** | **3.7** | 0.4 |
| SPEECH THERAPY: K | 3 | 2.2 | 4 | 4.5 | 2.7 | 3.7 | 3.4 | 4.2 | **3.3** | **3.7** | 0.4 |
| SPEECH THERAPY: L | 3 | 2.2 | 4 | 4.5 | 2.7 | 3.7 | 3.4 | 4.2 | **3.3** | **3.7** | 0.4 |
| SPEECH THERAPY: M | 3 | 2.2 | 4 | 4.5 | 2.7 | 3.7 | 3.4 | 4.2 | **3.3** | **3.7** | 0.4 |
| SPEECH THERAPY: N | 3 | 2.2 | 4 | 4.5 | 2.7 | 3.7 | 3.4 | 4.2 | **3.3** | **3.7** | 0.4 |
| SPEECH THERAPY: P | 3 | 2.2 | 4 | 4.5 | 2.7 | 3.7 | 3.4 | 4.2 | **3.3** | **3.7** | 0.4 |
| SPEECH THERAPY: R | 3 | 2.2 | 4 | 4.5 | 2.7 | 3.7 | 3.4 | 4.2 | **3.3** | **3.7** | 0.4 |
| SPEECH THERAPY: S PRACTICE | 2.2 | 2.4 | 3.8 | 4.5 | 2 | 2.7 | 3 | 3.3 | **2.8** | **3.2** | 0.4 |
| SPEECH THERAPY: S | 3 | 2.2 | 4 | 4.5 | 2.7 | 3.7 | 3.4 | 4.2 | **3.3** | **3.7** | 0.4 |
| SPEECH THERAPY: SH | 3 | 2.2 | 4 | 4.5 | 2.7 | 3.7 | 3.4 | 4.2 | **3.3** | **3.7** | 0.4 |
| SPEECH THERAPY: T | 3 | 2.2 | 4 | 4.5 | 2.7 | 3.7 | 3.4 | 4.2 | **3.3** | **3.7** | 0.4 |
| SPEECH THERAPY: TH | 3 | 2.2 | 4 | 4.5 | 2.7 | 3.7 | 3.4 | 4.2 | **3.3** | **3.7** | 0.4 |
| SPEECH THERAPY: V | 3 | 2.2 | 4 | 4.5 | 2.7 | 3.7 | 3.4 | 4.2 | **3.3** | **3.7** | 0.4 |
| SPEECH THERAPY: Z | 3 | 2.2 | 4 | 4.5 | 2.7 | 3.7 | 3.4 | 4.2 | **3.3** | **3.7** | 0.4 |
| SPEECH TRAINER 3D | 3.2 | 2.8 | 4.5 | 4.8 | 4.3 | 4 | 3.3 | 3.7 | **3.8** | **3.8** | 0 |
| SPEECHTUTORFREE | 3 | 2.4 | 3.3 | 4.3 | 4.3 | 3.7 | 3.7 | 3.3 | **3.6** | **3.4** | 0.2 |
| SPEECH WITH MILO ARTICULATION BOARD GAME PRO | 4.4 | 4 | 4.8 | 4.5 | 5 | 5 | 3.7 | 3.7 | **4.5** | **4.3** | 0.2 |
| SPEECHBOX FOR ARTICULATION SPEECH THERAPY-IPAD EDITION | 3.4 | 3.4 | 4.3 | 4.8 | 4.7 | 4.3 | 3 | 3.7 | **3.9** | **4** | 0.1 |
| SPEECHBOX FOR SPEECH THERAPY (APRAXIA, AUTISM, DOWN’S SYNDROME) IPHONE EDITION | 3.4 | 3.4 | 4.5 | 4.5 | 4.3 | 4.3 | 3.7 | 3.7 | **4** | **4** | 0 |
| STS ARTIC. TOWN L | 3.6 | 3.6 | 3 | 4 | 3.3 | 4.3 | 3.7 | 4 | **3.4** | **4** | 0.6 |
| STS ARTIC. TOWN S | 3.6 | 3.6 | 3 | 4 | 3.3 | 4.3 | 3.7 | 4 | **3.4** | **4** | 0.6 |
| TALKIE ARTICULATION | 4.2 | 4.2 | 4.5 | 4.5 | 3.7 | 5 | 3.7 | 3.7 | **4** | **4.4** | 0.4 |
| THE R APP | 2.8 | 2.8 | 3 | 4 | 2.3 | 3.3 | 2.3 | 3.7 | **2.6** | **3.5** | 0.9 |
| TIC-TAC-TALK | 3.2 | 3 | 3.8 | 4.5 | 2.7 | 3.7 | 3.3 | 3.7 | **3.3** | **3.7** | 0.4 |
| TIGA TALK SPEECH THERAPY GAMES | 3.2 | 3.2 | 4.5 | 4.5 | 4.3 | 4.3 | 3.3 | 3.3 | **3.8** | **3.8** | 0 |
| WACKY SELFIE ARTICULATION FOR SPEECH THERAPY | 3.6 | 3 | 5 | 4.5 | 4.7 | 4.3 | 3.3 | 3.3 | **4.2** | **3.8** | 0.4 |
| WEBBER PHOTO ARTIC CASTLE PRO | 4 | 4.6 | 4 | 4.5 | 4.3 | 4.7 | 4 | 4 | **4.1** | **4.5** | 0.4 |
| WHAT’S THE PIC ARTICULATION | 3.8 | 3 | 4 | 4.8 | 4.3 | 4 | 4 | 3.7 | **4** | **3.9** | 0.1 |
| WORD FLIPS | 4 | 3.2 | 4 | 4.5 | 3.7 | 4 | 4 | 4 | **3.9** | **3.9** | 0 |
| WORD SEARCH ARTICULATION FOR SPEECH THERAPY | 3.4 | 3 | 4 | 4.8 | 4 | 4.3 | 3.3 | 3.3 | **3.7** | **3.9** | 0.2 |
