# Supplementary material for: Mobile apps for treatment of speech disorders in children: An evidence-based analysis of quality and efficacy
Source: PLoS One. 2018 Aug 9;13(8):e0201513. doi: 10.1371/journal.pone.0201513 (PMC6084897; doi:10.1371/journal.pone.0201513)
Supplement: S3 Appendix — (DOCX) [file pone.0201513.s003.docx]

**S3 Appendix: Individual app characteristics by rank order**

| **RANK ORDER BY MARS SCORE** | **APP** | **VERSION** | **PLATFORM:**  1= Android  2= Apple  3= both | **RELEASE DATE** | **# OF UPDATES** | **AVERAGE UPDATE FREQUENCY (MONTHS)** | **RATING CURRENT VERSION OUT OF 5** | **# OF CURRENT RATINGS** | **LITE VERSION PRICE (AUD)** | **FULL VERSION PRICE (AUD)** | **DEVELOPER:**  1= Commercial app developer  2= SLP  3= unknown | **AVERAGE TOTAL MARS SCORE** | **AVERAGE MARS STAR RATING** | **AVERAGE MARS BEHAVIOUR CHANGE** |
| --- | --- | --- | --- | --- | --- | --- | --- | --- | --- | --- | --- | --- | --- | --- |
| 1 | ARTICULATION STATION PRO | 2.2.3 | 2 | 04-Feb-12 | 15 | 4.1 |  |  | $0.00 | $89.99 | 2 | 4.6 | 5 | 4.5 |
| 2 | APRAXIA RAINBOWBEE | 1.7 | 2 | 14-Oct-13 | 8 | 5.1 |  |  |  | $41.99 | 2 | 4.45 | 4.5 | 4.5 |
| 3 | ARTICULATION SCENES | 1.7 | 2 | 30-May-12 | 6 | 9.7 |  |  |  | $46.99 | 2 | 4.45 | 5 | 4.5 |
| 4 | ARTICULATE IT! PRO | 4.5 | 2 | 08-Dec-11 | 24 | 2.6 |  |  |  | $67.99 | 2 | 4.4 | 5 | 4.5 |
| 5 | SPEECH WITH MILO ARTICULATION BOARD GAME PRO | 1.2 | 2 | 13-Mar-13 | 2 | 24 |  |  | $10.99 | $37.99 | 2 | 4.4 | 5 | 4.5 |
| 6 | ARTICULATION VACATION | 1.7 | 2 | 29-Oct-14 | 7 | 4.1 |  |  |  | $46.99 | 2 | 4.3 | 4.5 | 4 |
| 7 | WEBBER PHOTO ARTIC CASTLE PRO | 1.2 | 2 | 07-Oct-12 | 5 | 10.6 |  |  | $0.00 | $62.99 | 2 | 4.3 | 4.5 | 4 |
| 8 | ARTICULATION CARNIVAL PRO | 1.5 | 3 | 06-Jan-14 | 4 | 9.5 |  |  | $0.00 | $57.99 | 2 | 4.25 | 4 | 4 |
| 9 | PHONOLOGICAL PROCESSES | 2.0.1 | 2 | 06-Mar-13 | 5 | 9.6 |  |  |  | $26.99 | 2 | 4.25 | 4 | 4.5 |
| 10 | SPEECH ESSENTIALS THERAPY APP | 1.39 | 1 |  |  |  | 3.4 | 112 | $0.00 | $49.99 | 2 | 4.2 | 4 | 4 |
| 11 | TALKIE ARTICULATION | 2.0.0 | 2 | 17-Oct-14 | 5 | 5.8 |  |  | $0.00 | $55.99 | 3 | 4.2 | 4.5 | 4 |
| 12 | ARTICULATION GAMES | 2.3 | 2 | 21-Sep-12 | 10 | 5.4 |  |  | $0.00 | $54.99 | 2 | 4.15 | 4.5 | 4 |
| 13 | FUN WITH R | 1.3 | 2 | 10-Sep-13 | 4 | 10.5 |  |  |  | $22.99 | 2 | 4.15 | 4 | 4 |
| 14 | APRAXIA PICTURE SOUND CARDS APSC | 2 | 2 | 06-Jul-11 | 3 | 22.7 |  |  | $139.99 | $279.99 | 3 | 4.1 | 4 | 5 |
| 15 | R INTENSIVE PRO | 4 | 2 | 12-May-10 | 5 | 16.4 |  |  |  | $30.99 | 2 | 4.1 | 3.5 | 3.5 |
| 16 | KIDS SOUND LAB- D SOUND OF THE WOODPECKER | 2.5 | 2 | 09-Aug-13 | 9 | 4.8 |  |  | $0.00 | $30.99 | 2 | 4.05 | 4 | 4 |
| 17 | MINIMAL PAIRS ACADEMY | 4 | 2 | 09-Feb-12 | 6 | 10.2 |  |  |  | $30.99 | 2 | 4 | 3.5 | 3.5 |
| 18 | SCIP (SOUND CONTRASTS IN PHONOLOGY) | 1.0.26 | 2 | 01-Nov-16 | 4 | 1 |  |  |  | $259.99 | 2 | 4 | 4 | 4 |
| 19 | SPEECHBOX FOR SPEECH THERAPY (APRAXIA, AUTISM, DOWN’S SYNDROME)- IPHONE EDITION | 1.1.2 | 2 | 04-Feb-13 | 2 | 24.5 |  |  |  | $14.99 | 1 | 4 | 4 | 4 |
| 20 | WACKY SELFIE ARTICULATION FOR SPEECH THERAPY | 1.0.1 | 2 | 23-Oct-16 | 1 | 5 |  |  |  | $7.99 | 2 | 4 | 3.5 | 3 |
| 21 | LEXICO ARTICULATION | 1 | 2 | 27-Jun-16 | 0 | 0 |  |  | $0.00 | $46.99 | 2 | 3.95 | 3.5 | 3.5 |
| 22 | MINIMAL PAIRS (THERAPY BOX LIMITED) | 2 | 2 | 09-Sep-11 | 7 | 9.4 |  |  |  | $5.99 | 3 | 3.95 | 4 | 4 |
| 23 | SPEECH THAT WORKS | 1.0.1 | 3 |  |  |  | 5 | 1 |  | $51.99 | 2 | 3.95 | 3.5 | 4 |
| 24 | SPEECHBOX FOR ARTICULATION SPEECH THERAPY-IPAD EDITION | 2.0.4 | 2 | 24-Oct-12 | 9 | 5.9 |  |  | $30.99 | $29.90 | 1 | 3.95 | 3.5 | 3.5 |
| 25 | WHAT’S THE PIC ARTICULATION | 1.1 | 2 | 30-Aug-16 | 1 | 7 |  |  |  | $7.99 | 2 | 3.95 | 3.5 | 3 |
| 26 | ARTICULATION LITE WITH THE SPEECH WIZARD | 1 | 2 | 06-Nov-15 | 0 | 0 |  |  | $0.00 | $55.99 | 2 | 3.9 | 3.5 | 3.5 |
| 27 | ARTICULATION FLIP BOOKS FREE | 1.6 | 2 | 13-Aug-13 | 2 | 21.5 |  |  | $0.00 | $119.99 | 1 | 3.9 | 3 | 3 |
| 28 | ARTIKPIX | 2.5.1 | 2 | 01-Sep-10 | 16 | 4.9 |  |  | $0.00 | $46.99 | 2 | 3.9 | 3 | 3 |
| 29 | CHARADES ARTICULATION FOR SPEECH THERAPY | 1.0.2 | 2 | 08-Apr-14 | 2 | 17.5 |  |  |  | $7.99 | 2 | 3.9 | 3.5 | 3 |
| 30 | MY ARTICULATION: INITIAL K | 2.1 | 2 | 08-Jan-16 | 2 | 7 |  |  |  | $9.99 | 2 | 3.9 | 3.5 | 3 |
| 31 | PUPPY MINIMAL PAIRS | 1.02 | 2 | 21-Jul-15 | 0 | 0 |  |  | $0.00 | $26.99 | 2 | 3.9 | 3.5 | 3.5 |
| 32 | WORD FLIPS | 1.0.3 | 2 | 29-Oct-13 | 3 | 13.7 |  |  |  | $46.99 | 2 | 3.9 | 4 | 3.5 |
| 33 | ARTIKPIX LEVELS | 1.0.3 | 2 | 06-Nov-14 | 3 | 9.7 |  |  | $0.00 | $46.99 | 2 | 3.85 | 3 | 3 |
| 34 | CLUSTERS COMPLEX LITE | 1.1.0 | 2 | 02-Jun-14 | 1 | 33 |  |  | $0.00 | $38.99 | 2 | 3.85 | 2.5 | 3 |
| 35 | GAME SHOW ARTICULATION FOR SPEECH THERAPY | 1 | 2 | 26-Feb-15 | 0 | 0 |  |  |  | $7.99 | 2 | 3.85 | 3 | 3 |
| 36 | MISSING LETTER ARTICULATION FOR SPEECH THERAPY | 1.0.1 | 2 | 03-Feb-15 | 1 | 25 |  |  |  | $7.99 | 2 | 3.85 | 3 | 2.5 |
| 37 | SECRET MISSION ARTICULATION FOR SPEECH THERAPY | 1.3.0 | 2 | 24-Feb-14 | 3 | 12.3 |  |  |  | $7.99 | 2 | 3.85 | 3 | 2.5 |
| 38 | SILLY SENTENCE ARTICULATION FOR SPEECH THERAPY | 1.3.0 | 2 | 07-Nov-14 | 2 | 14 |  |  |  | $7.99 | 2 | 3.85 | 3 | 3 |
| 39 | DANCE PARTY ARTICULATION FOR SPEECH THERAPY | 1.1.0 | 2 | 21-Apr-16 | 1 | 11 |  |  |  | $7.99 | 2 | 3.8 | 3 | 3 |
| 40 | ELR-OFFLINE FOR IPAD | 1.7.030 | 2 | 23-Mar-15 | 24 | 1 |  |  | $0.00 | $100 | 2 | 3.8 | 3 | 3 |
| 41 | I DARE YOU ARTICULATION FOR SPEECH THERAPY | 1.1.1 | 2 | 03-May-13 | 3 | 15.3 |  |  |  | $7.99 | 2 | 3.8 | 3 | 3 |
| 42 | MULTIPLE CHOICE ARTICULATION FOR SPEECH THERAPY | 1.1.1 | 2 | 27-Nov-12 | 5 | 10.4 |  |  |  | $7.99 | 2 | 3.8 | 3 | 3 |
| 43 | OPEN-ENDED ARTICULATION FOR SPEECH THERAPY | 1.1.1 | 2 | 18-Sep-13 | 2 | 21 |  |  |  | $7.99 | 2 | 3.8 | 3 | 3 |
| 44 | SPEECH TRAINER 3D | 3 | 2 | 02-Apr-11 | 6 | 11.8 |  |  |  | $12.99 | 2 | 3.8 | 3 | 3 |
| 45 | TIGA TALK SPEECH THERAPY GAMES | 1.3 | 2 | 29-Dec-10 | 3 | 25 | 4 | 5 |  | $7.99 | 2 | 3.8 | 3 | 3 |
| 46 | WORD SEARCH ARTICULATION FOR SPEECH THERAPY | 1.2.0 | 2 | 20-Jun-14 | 2 | 16.5 |  |  |  | $7.99 | 2 | 3.8 | 3 | 3 |
| 47 | ARTIC PHOTOS ‘L’ FUN DECK PLUS | 3.8 | 2 | 22-May-14 | 1 | 34 |  |  |  | $5.99 | 2 | 3.75 | 3 | 3 |
| 48 | ARTIC PHOTOS ‘R’ FUN DECK PLUS | 3.8 | 2 | 22-May-14 | 1 | 34 |  |  |  | $5.99 | 2 | 3.75 | 3 | 3 |
| 49 | ARTIC PHOTOS ‘S’ FUN DECK PLUS | 3.8 | 2 | 22-May-14 | 1 | 34 |  |  |  | $5.99 | 2 | 3.75 | 3 | 3 |
| 50 | ARTICULATION GAME MDB | 1.1 | 3 | 04-Aug-14 | 1 | 31 |  |  |  | $1.49 | 2 | 3.75 | 2.5 | 2.5 |
| 51 | ARTICULATION R & R BLENDS | 1 | 3 | 18-Aug-14 | 0 | 0 |  |  |  | $4.49 | 2 | 3.75 | 2.5 | 2.5 |
| 52 | DRILLABY PRO SPEECH THERAPY GAME-SLP EDITION | 2 | 2 | 10-Apr-13 | 8 | 5.9 |  |  |  | $38.99 | 2 | 3.75 | 3.5 | 3 |
| 53 | DRILLABY -FAMILY EDITION | 2 | 2 | 25-Apr-13 | 7 | 6.7 |  |  | $0.00 | $49.95 | 2 | 3.75 | 3.5 | 3 |
| 54 | FRICATIVES ARTICULATION | 1.1 | 3 | 01-Jun-15 | 1 | 21 |  |  |  | $1.49 | 2 | 3.75 | 3 | 2.5 |
| 55 | L & L BLENDS | 1 | 3 | 06-Mar-15 | 0 | 0 |  |  |  | $2.99 | 2 | 3.75 | 3 | 2.5 |
| 56 | PHONICS STUDIO | 1 | 2 | 03-Aug-12 | 0 | 0 | 3 | 15 |  | $2.99 | 3 | 3.75 | 3 | 3 |
| 57 | S, Z, & S BLENDS | 1.6 | 2 | 30-Oct-14 | 3 | 9.7 |  |  |  | $4.49 | 2 | 3.75 | 3 | 2.5 |
| 58 | SPEECH FLIPBOOK- ARTICULATION & APRAXIA | 2 | 2 | 02-Jul-13 | 5 | 8.8 |  |  | $0.00 | $14.99 | 2 | 3.75 | 3.5 | 3.5 |
| 59 | FLEXIBLE SPEECH | 1.5.4 | 2 | 03-Sep-14 | 9 | 3.3 |  |  | $0.00 | $24.99 | 2 | 3.7 | 3.5 | 3.5 |
| 60 | FLEXIBLE SPEECH USA | 1.1 | 2 | 20-Jan-16 | 1 | 14 |  |  | $0.00 | $14.99 | 2 | 3.7 | 3.5 | 3.5 |
| 61 | GHK ARTICULATION | 1 | 3 | 02-May-15 | 0 | 0 |  |  |  | $4.49 | 2 | 3.7 | 3 | 2.5 |
| 62 | LINGUISYSTEMS PHONOLOGY CARDS | 1 | 2 | 06-Apr-13 | 0 | 0 |  |  | $0.00 | $38.99 | 2 | 3.7 | 3.5 | 3.5 |
| 63 | LISTEN CLOSE ARTICULATION FOR SPEECH THERAPY | 1.0.1 | 2 | 23-Nov-13 | 1 | 40 |  |  |  | $7.99 | 2 | 3.7 | 2.5 | 3 |
| 64 | MINIMAL PAIRS FOR SPEECH THERAPY | 1 | 2 | 22-Feb-17 | 0 | 0 |  |  |  | $1.49 | 3 | 3.7 | 3.5 | 3.5 |
| 65 | R ARTICULATION | 1.4 | 3 | 26-Feb-15 | 3 | 8.3 |  |  |  | $7.99 | 2 | 3.7 | 2.5 | 2.5 |
| 66 | SH CH ARTICULATION | 1 | 3 | 14-May-15 | 0 | 0 |  |  |  | $4.49 | 2 | 3.7 | 2.5 | 2.5 |
| 67 | SPEECH SOUNDS FOR KIDS- AUSTRALIAN EDITION | 1 | 2 | 24-Feb-14 | 0 | 0 |  |  | $0.00 | $19.99 | 1 | 3.7 | 4 | 3.5 |
| 68 | SPEECH SOUNDS FOR KIDS LITE-US EDITION | 1 | 2 | 24-Feb-14 | 0 | 0 |  |  | $0.00 | $19.99 | 1 | 3.7 | 4 | 3.5 |
| 69 | STS ARTIC. TOWN L | 1.0.2 | 1 |  |  |  |  |  |  | $4.08 | 2 | 3.7 | 3.5 | 3 |
| 70 | STS ARTIC. TOWN S | 1.0.2 | 1 |  |  |  | 2 | 1 |  | $3.85 | 2 | 3.7 | 3.5 | 3 |
| 71 | POCKET PAIRS | 1.3 | 2 | 02-Feb-11 | 4 | 18.3 |  |  |  | $30.99 | 2 | 3.65 | 3 | 3.5 |
| 72 | SPEECH CARDS BY TEACH APPS- FOR SPEECH THERAPY | 1.1 | 2 | 03-Nov-15 | 1 | 16 |  |  |  | $2.99 | 2 | 3.65 | 2.5 | 2.5 |
| 73 | SPEECH THERAPY CENTER | 1.01 | 2 | 07-Oct-14 | 1 | 29 |  |  |  | $5.99 | 2 | 3.65 | 3.5 | 3 |
| 74 | APRAXIA-EARLY INTERVENTION 1 | 1.1 | 1 | 10-Oct-10 | 1 | 77 |  |  | $0.00 | $2.99 | 3 | 3.6 | 3 | 3 |
| 75 | ARTICULATION FLASH CARDS /R/ | 1 | 1 |  |  |  | 3 | 4 |  | $3.02 | 3 | 3.6 | 2.5 | 2.5 |
| 76 | HIGH FREQUENCY WORDS FOR SPEECH THERAPY- FOR SPEECH THERAPY | 1.1 | 2 | 12-Oct-15 | 1 | 17 |  |  |  | $1.49 | 2 | 3.6 | 3 | 3 |
| 77 | I CAN ARTICULATE | 6.1.0 | 2 | 06-Apr-12 | 13 | 4.5 |  |  | $0.00 | $62.99 | 2 | 3.6 | 3 | 3 |
| 78 | S&S BLENDS ARTICULATION | 1.2 | 1 |  |  |  | 2.3 | 3 |  | $3.80 | 2 | 3.6 | 3 | 2 |
| 79 | POCKET ARTIC | 2.54 | 2 | 08-Mar-10 | 16 | 5.25 |  |  |  | $30.99 | 2 | 3.6 | 3 | 3.5 |
| 80 | APRAXIMATIONS | 1 | 2 | 10-Oct-15 | 0 | 0 |  |  |  | $0.00 | 2 | 3.55 | 2.5 | 2.5 |
| 81 | ARTICULATION COACH-L | 1 | 1 |  |  |  | 5 | 1 |  | $0.99 | 1 | 3.55 | 3 | 3 |
| 82 | ARTICULATION COACH-P | 1 | 1 |  |  |  | 3.5 | 34 |  | $0.00 | 1 | 3.55 | 3 | 3 |
| 83 | ARTICULATION COACH-R | 1 | 1 |  |  |  | 3 | 4 |  | $0.99 | 1 | 3.55 | 3 | 3 |
| 84 | JUNGANEW: A HERD OF SOUND S ‘S’ FREE | 1.1.3 | 2 | 03-Dec-15 | 1 | 15 |  |  | $0.00 | $14.99 | 2 | 3.55 | 3 | 2.5 |
| 85 | PHONOPIX-FULL | 2.1.1 | 2 | 21-Feb-11 | 2 | 36.5 |  |  |  | $46.99 | 2 | 3.55 | 2.5 | 3 |
| 86 | QUICK ARTIC | 2 | 2 | 10-Aug-11 | 6 | 11.2 |  |  |  | $0.00 | 2 | 3.55 | 3 | 3 |
| 87 | SPEECH THERAPY FOR APRAXIA-2 SYLLABLE WORDS | 1.2.1 | 2 | 03-Dec-13 | 3 | 13 |  |  | $7.99 | $7.99 | 1 | 3.55 | 3 | 3.5 |
| 88 | SPEECH THERAPY FOR APRAXIA –NACD SPEECH THERAPIST | 1.5.2 | 3 | 28-Mar-12 | 10 | 6 |  |  | $7.99 | $7.99 | 1 | 3.55 | 3 | 3.5 |
| 89 | SPEECH THERAPY FOR APRAXIA- WORDS | 1.2 | 3 | 22-Dec-12 | 2 | 25.5 |  |  |  | $7.99 | 1 | 3.55 | 3 | 3.5 |
| 90 | SPEECH THERAPY FOR APRAXIA-ENDINGS | 1.2.1 | 2 | 11-Feb-14 | 3 | 12.3 |  |  | $7.99 | $7.99 | 1 | 3.55 | 3 | 3.5 |
| 91 | ARTIC PRACTICE | 1.8 | 2 | 11-May-12 | 5 | 11.6 |  |  |  | $14.99 | 2 | 3.5 | 3 | 3.5 |
| 92 | ARTICULATION IV | 1.8 | 2 | 12-Apr-12 | 5 | 11.8 |  |  |  | $14.99 | 2 | 3.5 | 3 | 3.5 |
| 93 | GUESS THE PICTURE FOR ARTICULATION PRACTICE (CH, SH) | 1 | 2 | 20-Dec-13 | 0 | 0 |  |  |  | $4.49 | 2 | 3.5 | 3.5 | 3.5 |
| 94 | GUESS THE PICTURE FOR ARTICULATION PRACTICE (K, G) | 1 | 2 | 07-Jul-13 | 0 | 0 |  |  |  | $4.49 | 2 | 3.5 | 3.5 | 3.5 |
| 95 | SPEECH CORNERS | 1.4.1 | 2 | 20-Jan-11 | 5 | 14.8 |  |  | $5.99 | $89.99 | 2 | 3.5 | 2 | 2.5 |
| 96 | SPEECH HANGMAN | 1.4.1 | 2 | 18-Jan-11 | 5 | 14.8 |  |  | $7.99 | $89.99 | 2 | 3.5 | 2 | 2.5 |
| 97 | SPEECH SQUARES | 1.3.1 | 2 | 18-Jan-11 | 4 | 18.5 |  |  |  | $7.99 | 2 | 3.5 | 2 | 2.5 |
| 98 | SPEECH THERAPY: B | 1 | 1 |  |  |  |  |  |  | $2.69 | 2 | 3.5 | 4 | 3.5 |
| 99 | SPEECH THERAPY: CH | 1 | 1 |  |  |  |  |  |  | $2.69 | 2 | 3.5 | 4 | 3.5 |
| 100 | SPEECH THERAPY: D | 1 | 1 |  |  |  |  |  |  | $3.89 | 2 | 3.5 | 4 | 3.5 |
| 101 | SPEECH THERAPY: F | 1 | 1 |  |  |  | 1 | 1 |  | $3.99 | 2 | 3.5 | 4 | 3.5 |
| 102 | SPEECH THERAPY: G | 1 | 1 |  |  |  |  |  |  | $3.89 | 2 | 3.5 | 4 | 3.5 |
| 103 | SPEECH THERAPY: J | 1 | 1 |  |  |  |  |  |  | $3.99 | 2 | 3.5 | 4 | 3.5 |
| 104 | SPEECH THERAPY: K | 1 | 1 |  |  |  | 4 | 1 |  | $0.00 | 2 | 3.5 | 4 | 3.5 |
| 105 | SPEECH THERAPY: L | 1.02 | 1 |  |  |  | 3.1 | 8 |  | $0.00 | 2 | 3.5 | 4 | 3.5 |
| 106 | SPEECH THERAPY: M | 1 | 1 |  |  |  |  |  |  | $2.59 | 2 | 3.5 | 4 | 3.5 |
| 107 | SPEECH THERAPY: N | 1 | 1 |  |  |  |  |  |  | $3.99 | 2 | 3.5 | 4 | 3.5 |
| 108 | SPEECH THERAPY: P | 1 | 1 |  |  |  |  |  |  | $2.69 | 2 | 3.5 | 4 | 3.5 |
| 109 | SPEECH THERAPY: R | 1 | 1 |  |  |  | 1 | 1 |  | $3.96 | 2 | 3.5 | 4 | 3.5 |
| 110 | SPEECH THERAPY: S | 1 | 1 |  |  |  |  |  |  | $3.96 | 2 | 3.5 | 4 | 3.5 |
| 111 | SPEECH THERAPY: SH | 1 | 1 |  |  |  |  |  |  | $3.99 | 2 | 3.5 | 4 | 3.5 |
| 112 | SPEECH THERAPY: T | 1 | 1 |  |  |  |  |  |  | $3.89 | 2 | 3.5 | 4 | 3.5 |
| 113 | SPEECH THERAPY: TH | 1 | 1 |  |  |  |  |  |  | $4.09 | 2 | 3.5 | 4 | 3.5 |
| 114 | SPEECH THERAPY: V | 1 | 1 |  |  |  |  |  |  | $3.89 | 2 | 3.5 | 4 | 3.5 |
| 115 | SPEECH THERAPY: Z | 1 | 1 |  |  |  |  |  |  | $3.89 | 2 | 3.5 | 4 | 3.5 |
| 116 | SPEECHTUTORFREE | 2.04 | 2 | 06-Sep-14 | 3 | 10 |  |  | $0.00 | $22.99 | 2 | 3.5 | 3.5 | 3 |
| 117 | TIC-TAC-TALK | 1.5.2 | 2 | 21-Dec-10 | 7 | 10.7 |  |  | $7.99 | $89.99 | 2 | 3.5 | 2 | 2.5 |
| 118 | DIADO SYLLABLES | 1.3 | 3 | 17-Nov-14 | 3 | 9.3 |  |  | $0.00 | $5.99 | 2 | 3.45 | 1.5 | 1.5 |
| 119 | SPEECH SOUNDS ON CUE FOR IPAD LITE (AUS ENGLISH) | 1.3 | 2 | 18-Oct-11 | 3 | 21.7 |  |  | $0.00 | $19.99 | 1 | 3.45 | 2.5 | 2.5 |
| 120 | PHONOLOGY MATCHUPS! | 1.8 | 2 | 07-Apr-15 | 0 | 0 |  |  |  | $2.99 | 2 | 3.4 | 2.5 | 2.5 |
| 121 | LISP THERAPY FREE | 1.02 | 2 | 10-Apr-15 | 1 | 23 |  |  | $0.00 | $38.99 | 1 | 3.35 | 2 | 2.5 |
| 122 | SLP MINIMAL PAIRS LITE-TOOL FOR SPEECH THERAPY | 2.5.0 | 2 | 17-Mar-11 | 10 | 7.2 |  |  | $0.00 | $46.99 | 2 | 3.35 | 2.5 | 2.5 |
| 123 | MINIMAL PAIRS (LEARNING FUNDAMENTALS) | 1.8 | 2 | 09-Mar-12 | 7 | 8.6 |  |  |  | $9.99 | 2 | 3.3 | 2 | 2.5 |
| 124 | PHONOLOGY | 1.8 | 2 | 24-May-12 | 4 | 14.5 |  |  |  | $9.99 | 2 | 3.3 | 2 | 2.5 |
| 125 | BIGMOUTH SOUNDS | 2.1 | 2 | 16-Jan-12 | 2 | 31 |  |  |  | $7.99 | 3 | 3.2 | 2 | 1 |
| 126 | MYARTIC | 2.0.0 | 2 | 14-Nov-12 | 1 | 52 |  |  | $0.00 | $9.99 | 3 | 3.2 | 2.5 | 2.5 |
| 127 | SPEECH CARDS LITE | 1.4.0 | 2 | 05-Nov-12 | 8 | 6.5 |  |  | $0.00 | $14.99 | 3 | 3.2 | 2 | 2 |
| 128 | SPEECH THERAPY, APRAXIA ARTICULATION WORD FLASHCARDS | 1 | 3 | 13-Aug-15 | 0 | 0 |  |  | $0.00 | $14.99 | 3 | 3.15 | 2.5 | 2.5 |
| 129 | THE R APP | 1 | 2 | 18-Aug-11 | 0 | 0 |  |  |  | $7.99 | 2 | 3.05 | 1.5 | 2 |
| 130 | SPEECH THERAPY: S PRACTICE | 3 | 1 |  |  |  | 3.3 | 48 |  | $0.00 | 3 | 3 | 2 | 2 |
| 131 | ARTICULATION ISLAND | 1.2.3 | 2 | 07-May-14 | 5 | 6.8 |  |  |  | $5.99 | 3 | 2.8 | 1.5 | 2 |
| 132 | ALL ABOUT SOUNDS HD-INITIAL POSITION WORDS LITE | 1.9.105 | 2 |  |  |  | 3.2 | 12 | $0.00 | $0.00 | 1 | 2.45 | 1.5 | 1.5 |
