# Supplementary material for: Mobile apps for treatment of speech disorders in children: An evidence-based analysis of quality and efficacy
Source: PLoS One. 2018 Aug 9;13(8):e0201513. doi: 10.1371/journal.pone.0201513 (PMC6084897; doi:10.1371/journal.pone.0201513)
Supplement: S4 Appendix — (DOCX) [file pone.0201513.s004.docx]

| **APP TITLE AND BEHAVIOURAL CHANGE SCORE** | **INTERVENTION PRINCIPLES [1]** | | | |
| --- | --- | --- | --- | --- |
|  | **PHONOLOGY**  Focus is on learning the rules of the phonological system. Intervention is meaning-based and uses targets which facilitate widespread change in the phonological system. | **MOTOR LEARNING**  Learning which focusses on speech production within pre-practice and practice phases, with careful and calculated consideration of the conditions of practice and feedback. | **SPEECH PERCEPTION**  Focus is on listening to spoken language and perceiving differences between phonemes in varying contexts and speakers. Opportunities for judging speech production accuracy are provided. | **COGNITION AND META-AWARENESS**  Focus is on how the user perceives, creates and retrieves phonological representations by developing the user’s phonetic, phonological, prosodic and pragmatic awareness. Self-evaluation and metaphors are used to support learning. |
| APRAXIA PICTURE SOUND CARDS APSC (5) |  | ☑ |  |  |
| ARTICULATION STATION PRO (4.5) |  | ☑ |  |  |
| APRAXIA RAINBOWBEE (4.5) |  | ☑ |  |  |
| ARTICULATION SCENES (4.5) |  | ☑ |  |  |
| ARTICULATE IT! PRO (4.5) |  | ☑ |  |  |
| SPEECH WITH MILO ARTICULATION BOARD GAME PRO (4.5) |  | ☑ |  |  |
| PHONOLOGICAL PROCESSES (4.5) | ☑ |  |  |  |
| SPEECH ESSENTIALS THERAPY APP (4) |  | ☑ |  |  |
| ARTICULATION VACATION (4) |  | ☑ |  |  |
| WEBBER PHOTO ARTIC CASTLE PRO (4) |  | ☑ |  |  |
| TALKIE ARTICULATION (4) |  | ☑ |  |  |
| ARTICULATION GAMES (4) |  | ☑ |  |  |
| FUN WITH R (4) |  | ☑ |  |  |
| KIDS SOUND LAB- D SOUND OF THE WOODPECKER (4) |  | ☑ |  | ☑ |
| SCIP (SOUND CONTRASTS IN PHONOLOGY) (4) |  | ☑ |  |  |
| SPEECHBOX FOR SPEECH THERAPY (APRAXIA, AUTISM, DOWN SYNDROME) (IPHONE EDITION) (4) |  | ☑ |  |  |
| MINIMAL PAIRS (THERAPY BOX LIMITED) (4) |  | ☑ |  |  |
| ARTICULATION CARNIVAL PRO (4) |  | ☑ |  |  |
| SPEECH THAT WORKS (4) |  | ☑ |  |  |

**S4 Appendix: Therapeutic features of top 19 apps for behaviour change**

| **APP TITLE AND BEHAVIOURAL CHANGE SCORE** | **INTERVENTION PROCEDURES [1]** | | | | | |
| --- | --- | --- | --- | --- | --- | --- |
|  | **ANTECEDENT INSTRUCTION**  The first part of the teaching moment: learning cues, actions or instructions provided prior to the user’s response encouraging listening (auditory cue) or to elicit speech (production cue). | **USER RESPONSE**  The second part of the teaching moment: expected or anticipated response/s from the user. | **CONSEQUENT EVENT**  The third part of the teaching moment: feedback, reinforcement or learning cues following the user’s response. | **INTERVENTION STIMULUS**  The skill being practised or targeted in the teaching moment; the therapy exemplars. | **INTERVENTION RESOURCE**  Pictures or objects used to elicit the stimuli. | **INTERVENTION ACTIVITY**  Tasks used to motivate or engage the user in the intervention. |
| APRAXIA PICTURE SOUND CARDS APSC (5) | Production and auditory cues. | Imitation or spontaneous speech production, looking at visual information while producing speech, phonological awareness tasks including grapheme/phoneme correspondence, auditory judgement of own speech accuracy and self-correction. | Reinforcement sound buttons. | Isolated phonemes and phonemes at word, phrase and sentence level.  Consonant and vowel graphemes, hand cues for speech sounds.  Customisation for word position and syllable/sound structure.  All 22 consonant phonemes. Missing /ʒ, ð/.  18 vowel phonemes/all short vowels. | Written word or pictures. | Flashcards. |
| ARTICULATION STATION PRO (4.5) | Production and auditory cues. | Imitation or spontaneous speech production, reading, auditory judgement of own speech accuracy and self-correction. | Reinforcement button sounds, scoring tally, picture and sound effects for completed tasks, activity reinforcers (e.g. sound effects in matching game). | Word, phrase, sentence and story level.  Customisation for word position and syllable structure.  23 consonant phonemes. Missing /ʒ/. | Picture with corresponding written word. | Flashcards, matching game, creating sentences, stories with comprehension questions.  Ability to audio-record speech and self-evaluate. |
| APRAXIA RAINBOWBEE (4.5) | Production and auditory cues. | Imitation or spontaneous speech production, looking at visual information, auditory judgement of own speech accuracy and self-correction. | Reinforcement button sounds, scoring tally, tangible rewards (e.g. earning stickers), and activity reinforcers (e.g. progress in game). | Word and phrase level. Customisation for word position and syllable structure.  20 consonant phonemes (organised by place of articulation). Missing / ʒ, ŋ, j, z/. | Written words or pictures. | Flashcards and a board game.  Ability to audio-record speech and self-evaluate. |
| ARTICULATION SCENES (4.5) | Production and auditory cues. | Passive listening, imitation or spontaneous speech production, reading, auditory judgement of own speech accuracy and self-correction. | Reinforcement button sounds, scoring tally, picture/sound effects for completed tasks, tangible rewards (e.g. collectable accolades) and activity reinforcers (e.g. watching own movie productions). | Word or story level. Customisation for word position.  23 consonant phonemes. Missing /ʒ/. | Written words and/or pictures. | Finding hidden items, tap and say it, creating stories or retelling stories for picture scenes (the movie theatre/ the production room).  Ability to audio-record speech and self-evaluate. |
| ARTICULATE IT! PRO (4.5) | Production and auditory cues. | Passive listening, imitation or spontaneous speech production, reading, looking at visual information, auditory judgement of own speech accuracy and self-correction. | Reinforcement button sounds, scoring tally, activity reinforcer (e.g. sound effects in matching game). | Word, phrase, sentence and story level.  Customisation for word position and syllable structure.  23 consonant phonemes. Missing /ʒ/. | Written words and/or pictures. | Flashcards, matching game, stories with comprehension questions and a guessing game.  Ability to audio-record speech and self-evaluate. |
| SPEECH WITH MILO ARTICULATION BOARD GAME PRO (4.5) | Production cues. | Imitation or spontaneous speech production. | Activity reinforcers (e.g. sound effects/progress in game). | Word level. Customisation for word position.  22 consonant phonemes. Missing /ʒ, ð/. | Written words and/or pictures. | Board game. |
| PHONOLOGICAL PROCESSES (4.5) | Production and auditory cues. | Passive listening, spoken word recognition, auditory judgement of own speech accuracy, self-correction and imitation or spontaneous speech production. | Reinforcement button sounds, scoring tally, activity reinforcers (e.g. animations in game). | Words presented as minimal pairs. Customisation for target sounds in pairs.  8 phonological processes: affrication, deaffrication, cluster reduction, final consonant deletion, fronting, gliding, prevocalic voicing and stopping. | Written words and pictures. | Flashcards and board game.  Ability to audio-record speech and self-evaluate. |
| SPEECH ESSENTIALS THERAPY APP (4) | Production cues. | Imitation or spontaneous speech production, looking at visual information while producing speech (mirror feature). | Reinforcement button sounds, scoring tally, activity reinforcers (e.g. sound/picture effects in games). | Word, and sentence level.  Customisation for word position.  All 24 consonant phonemes. | Written words and pictures. | Flashcards, matching game, word find, puzzle, create words. |
| ARTICULATION VACATION (4) | Production and auditory cues. | Imitation or spontaneous speech production, auditory judgement of own speech accuracy and self-correction. | Reinforcement button sounds, scoring tally, activity reinforcers (e.g. animations in game). | Word, phrase, sentence and story level.  Customisation for word position.  21 consonant phonemes. Missing /ð, ŋ, ʒ/. | Written words and/or pictures. | Vacation photos, beach treasure hunt, parachuting and fishing.  Ability to audio-record speech and self-evaluate. |
| WEBBER PHOTO ARTIC CASTLE PRO (4) | Production and auditory cues. | Imitation or spontaneous speech production, auditory judgement of own speech accuracy and self-correction. | Reinforcement button sounds, activity reinforcers (e.g. animations in game). | Word, phrase and sentence level. Customisation for word position and syllable structure.  22 consonant phonemes. Missing /ŋ, ʒ/. | Written words and pictures. | Flashcards, matching and six arcade games: balloon pop, duck derby, fishing rodeo, hidden treasure, raceway and space explorer.  Ability to audio-record speech and self-evaluate. |
| TALKIE ARTICULATION(4) | Production and auditory cues. | Imitated speech production, auditory judgement of own speech accuracy and self-correction. | Reinforcement button sounds, scoring tally, activity reinforcers (e.g. animations in game), and picture/sound effects for completed tasks. | Word level.  Customisation for word position, semantic difficulty, articulation difficulty and syllable structure.  23 consonant phonemes. Missing /ʒ/. | Written words and pictures. | Flashcards presented in four different activities: racing car, balloon popping, trains, bricks/wrecking ball and image exposure.  Ability to audio-record speech and self-evaluate. |
| ARTICULATION GAMES (4) | Production and auditory cues. | Imitation or spontaneous speech production, auditory judgement of own speech accuracy and self-correction. | Reinforcement button sounds, scoring tally, activity reinforcers (e.g. animations in game). | Word, phrase and sentence level. Customisation for word position.  22 consonant phonemes. Missing /ŋ, ʒ/. | Written words and/or pictures. | Flashcards, memory game, articulation spinner and a pet wheel.  Ability to audio-record speech and self-evaluate. |
| FUN WITH R (4) | Production and auditory cues. | Imitation or spontaneous speech production, auditory judgement of own speech accuracy and self-correction. | Reinforcement button sounds, scoring tally, activity reinforcers (e.g. animations in game). | Word, phrase and sentence level. Customisation for word position.  Consonant phoneme /r/ including blends. | Written words and/or pictures. | Flashcards, memory game and bingo game.  Ability to audio-record speech and self-evaluate. |
| KIDS SOUND LAB- D SOUND OF THE WOODPECKER (4) | Production and auditory cues. | Imitated production, phonological awareness (initial sound identification  /sorting), spoken word recognition, auditory judgement of own speech accuracy and self-correction. | Picture/sound effects/animations for completed tasks. | Isolated phoneme, syllable and word level.  All 24 consonant phonemes. | Written words and pictures. | Animations, balloon popping, trains, picture sorting, matching.  Ability to audio-record speech and self-evaluate. |
| SCIP (SOUND CONTRASTS IN PHONOLOGY) (4) | Production and auditory cues. | Imitated, spontaneous or mastered production, auditory judgement of own speech accuracy and self-correction. | Change of colour of the border of completed cards, picture animation for correct production. | Word level in preselected sets according to elected treatment approach. Customisation for word lists, including use of nonsense words.  23 phonemes. Missing /ʒ/. | Written words and/or pictures. | Moveable flashcards.  Ability to audio-record speech and self-evaluate. |
| SPEECHBOX FOR SPEECH THERAPY (APRAXIA, AUTISM, DOWN SYNDROME) IPHONE EDITION (4) | Production and auditory cues. | Imitation or spontaneous speech production, auditory judgement of own speech accuracy and self-correction. | Activity reinforcers (e.g. emptying words from the speech box). | Word level. Customisation for word position.  20 consonant phonemes. Missing /ʒ, ð, ŋ, j/.  10 vocabulary categories for language therapy. | Written words and pictures. | Flashcards.  Ability to audio-record speech and self-evaluate. |
| MINIMAL PAIRS (THERAPY BOX LIMITED) (4) | Production and auditory cues. | Spoken word recognition, spontaneous production, auditory judgement of own speech accuracy and self-correction. | Reinforcement button sounds, picture/sound effects/animations for completed tasks and final score. | Words presented as minimal pairs. Customisation for target sounds in pairs. 21 consonant phonemes. Missing /ʒ, ð, ŋ/. | Written words and pictures. | Flashcards.  Ability to audio-record speech and self-evaluate. |
| ARTICULATION CARNIVAL PRO (4) | Production and auditory cues. | Imitation or spontaneous speech production, auditory judgement of own speech accuracy and self-correction. | Scoring tally, reinforcement button sounds and games after a set number of productions. | Word, phrase and sentence level. Customisation for word position.  22 consonant phonemes. Missing /ʒ, ŋ/. | Written words and/or pictures. | Flashcards with a choice of four reward games: spray ducks, basketball, balloon darts and a strength hammer.  Ability to audio-record speech and self-evaluate. |
| SPEECH THAT WORKS (4) | Production and auditory cues. | Passive listening, imitation or spontaneous speech production, auditory judgement of own speech accuracy and self-correction. | Scoring tally, activity reinforcers (e.g. progress in games). | Suprasegmentals (intensity, duration, pitch), consonants and vowels in isolation and at word, phrase and sentence level. Customisation for word position.  24 consonant phonemes.  13 vowel phonemes: 6 short vowels (/æ, ɛ, ɪ, ɒ, ʌ, ʊ/) and 7 long vowels (/i, u, aʊ, oʊ, aɪ, ɔɪ, eɪ/). | Written words and pictures. | Flashcards, matching and nursery rhymes.  Ability to audio-record speech and self-evaluate. |

1. McLeod S, Baker E. Children's speech: An evidence-based approach to assessment and intervention. Boston; USA: Pearson Education, Inc.; 2017.
